# Genomic information in the decision-making process for the training of a high-performance brazilian swimmer: a case report

**DOI:** 10.3389/fgene.2025.1544178

**Published:** 2025-04-15

**Authors:** Ricardo Muller Bottura, Daniel Blasioli Dentillo

**Affiliations:** ^1^ Academy - Health, Science and Performance, SãoPaulo, Brazil; ^2^ Department of Neurosciences, Biomedicine and Movement Sciences, Università degli Studi di Verona, Verona, Italy; ^3^ Department of Neuroscience, Rehabilitation, Ophthalmology, Genetics, and Maternal-Infant Sciences (DINOGMI), Università degli Studi di Genova, Genoa, Italy; ^4^ DGLab, Ribeirão Preto, Brazil

**Keywords:** genetics, swimming, high-performance, strength training, case report

## Abstract

Although numerous genetic variations have been associated with athletic profiles and performance, there is limited research on the real-world application of genetic insights in elite athlete training. The aim of this study is to present our 1-year training experience with a high-performing open water marathon swimmer, integrating genomic-based decision-making into training interventions. This case study involves a 23-year-old elite open water marathon swimmer whose primary goal was to qualify for the Absolute World Championships in 2024. The athlete had a consistent competitive history but sought optimized training strategies to enhance performance and secure a top position in national and international competitions. To personalize the training plan, twenty genetic polymorphisms were analyzed, guiding adjustments in strength training periodization and endurance capacity development. The interventions included tailored regimens aligned with the athlete’s genetic predispositions, aiming to maximize physiological responses, recovery, and performance. Additionally, longitudinal monitoring of training load was conducted to assess adaptation and optimize workload distribution. The outcome was an improvement in athletic performance, highlighted by a top finish among compatriots and qualification for the Absolute World Championships. This case report demonstrates that genetic-based training, when integrated with structured load monitoring, can be an effective strategy to assist sports professionals in planning and optimizing training for high-performance athletes. This approach enhances precision in training interventions, providing valuable support for decision-making in elite sports preparation.

## 1 Introduction

In April 2023, after finishing in the top 10 in the national qualifiers for the Open Water World Championships, the athlete contacted us to take care of his physical preparation out of the water (strength training). In addition, the athlete reported that he had a tendinopathy in the rotator cuff muscles of his left shoulder at the end of 2022 (small tears at the junction between the supraspinatus and infraspinatus tendons and a partial tear in the middle and lower fibers of the subscapularis tendon). This type of injury is not uncommon in swimming athletes (Gerrard, 1999). In fact, rotator cuff injuries are common in swimmers and overhead athletes. While the prevalence in the general population is around 20%, it may reach up to 40% among swimmers and overhead throwing athletes (Yamamoto et al., 2010; Connor et al., 2003). His body composition in January 2023 was 69.5 kg, with 17.4% body fat.

Our first decision was to perform a genetic test to look for polymorphisms related to athletic profile and performance. This is because the number of publications establishing a link between genetics and performance has increased every year since the Human Genome Project began (Ahmetov et al., 2016). Today, more than 250 polymorphisms have been associated with exercise and athletic performance (Semenova et al., 2023). Moreover, an estimated 50%–80% of athletic traits are considered heritable, with a point estimate of 66% on average (Semenova et al., 2023). Despite this, few studies have examined the use of this genetic information in long-term training interventions. Possible reasons that may help explain the lack of scientific work in high performance sport (Coutts, 2017) have been outlined by Buccheit (Buchheit, 2017) and this case study has a mission to help address this issue in relation to genetics in sport.

Among the various studies linking training to high performance, the recent review by Furrer et al. (2023) is perhaps the most significant, as it provides a comprehensive overview of the entire process that turns normal people into champions, including the genetic aspect. As suggested in two recent literature reviews (Kikuchi and Nakazato, 2015; Goodlin et al., 2015), we report how we used genetic information to modulate the acute training variables for our athlete with the aim of maximizing his physiological adaptations and minimizing injury risk.

The role of genetic polymorphisms in sports performance and injury risk has been extensively studied, with multiple research groups investigating associations between specific genetic variants and athletic traits (Ahmetov et al., 2016; Semenova et al., 2023). Previous studies have explored the relationship between genetic markers and physical attributes relevant to elite sports, including endurance capacity, muscle composition, injury susceptibility, and recovery efficiency (El Ouali et al., 2024; Ferreira et al., 2024; Vincent et al., 2007; Baumert et al., 2016). Notably, research efforts have employed genome-wide association studies (GWAS) and candidate gene approaches to identify potential predictors of performance and injury risk (Roos et al., 2017; Tanisawa et al., 2020).

However, while these studies provide a strong foundation for understanding the genetic contributions to athletic success, they have primarily been observational or predictive in nature, focusing on associations rather than the direct application of genetic information in personalized training interventions. To our knowledge, no prior study has actively implemented a genetically guided training program where training parameters are adjusted based on an athlete’s specific genetic profile. Our study represents a pioneering step in translating genomic data into practical applications by tailoring training loads, recovery protocols, and conditioning strategies according to the genetic predispositions of an elite athlete.

This study aims to bridge the gap between genetic research and its real-world application in high-performance sports by demonstrating how genetic information can be directly incorporated into a structured training program, thus optimizing athlete preparation and injury prevention strategies.

## 2 Brief case

### 2.1 Experimental design

This was a case study conducted on a high-performance athlete. The subject was a 23-year-old open water swimmer with a 10-year training history and consent to this case report. Before we started planning our training, we requested a sports genetics test from DGLab DNA Tests, Brazil. Using real-time PCR with the Taqman system, the company identified the athlete’s genotypes associated with the following genes and polymorphisms: ACTN3 (rs1815739), MCT1 (rs1049434), AGT (rs699), ACE (rs4341), BDKRB2 (rs1799722), ADRB2 (rs1042713), NOS3 (rs2070744 and rs1799983), PPARA (rs4253778), PPARD (rs20165209 and rs2267668), PPARGC1A (rs8192678), VEGF-A (rs2010963), TNF-A (rs1800629), IL-6 (rs1800795), CRP (rs1205), SOD2 (rs4880), CAT (rs1001179), GPX1 (rs1050450), and COL5A1 (rs12722). The genetic polymorphisms analyzed in this study were selected based on robust prior evidence of their association with athletic performance and physiological adaptations to training in accordance with Semenova et al. (Semenova et al., 2023). With this information, we categorized the genes according to their metabolic function and relationship to the targeted physiological adaptations to understand the athlete’s strengths and weaknesses (Table 1). The characteristics of each profile are explained in the Discussion.

**TABLE 1:** Genes selected for assessing the athlete’s trainability potential.

Genes	Genotypes	Athlete’s genotypes
TRAINABILITY PROFILE		
ACTN3 (rs1815739)	C/C (RR) and T/C (RX) - production of alpha-actinin-3 T/T (XX) - absence of alpha-actinin-3	CC
MCT1 (rs1049434)	A/A- higher production of MCT1 T/T and A/T - lower production of MCT1	AT
AGT (rs699)	C/C and T/C - higher production of angiotensinogen T/T - lower production of angiotensinogen	CC
ACE (rs4341)	G/G and C/G - higher production and action of ACE C/C - lower production and action of ACE	CC
BDKRB2 (rs1799722)	T/T - higher quantity of bradykinin receptor C/T and C/C - lower quantity of bradykinin receptor	CT
NOS3 (rs2070744)	T/T and C/T - higher production of NOS3 enzyme C/C - lower production of NOS3 enzyme	CC
NOS3 (rs1799983)	G/G - higher activity of NOS3 enzyme T/T and G/T - lower activity of NOS3 enzyme	GT
ADRB2 (rs1042713)	A/A- higher affinity of receptor to adrenaline A/G and G/G - lower affinity of receptor to adrenaline	GG
ADAPTATION PROFILE		
PPARA (rs4253778)	G/G and G/C - higher energy production from lipid metabolism C/C - lower energy production from lipid metabolism	CG
PPARD (rs20165209)	T/T and C/T - stimulates more PPARGC1A expression C/C - stimulates less PPARGC1A expression	CT
PPARD (rs2267668)	A/A and A/G - stimulates more PPARGC1A expression G/G - stimulates less PPARGC1A expression	AG
PPARGC1A (rs8192678)	C/C and C/T - higher production of mitochondria T/T - lower production of mitochondria	TT
VEGFA (rs2010963)	C/C and C/G - higher production of VEGF-A G/G - lower production of VEGF-A	GG
TNFA (rs1800629)	G/G - lower production of TNF-A A/A and A/G - higher production of TNF-A	GG
IL6 (rs1800795)	C/C - lower synthesis of IL-6 G/G and C/G - higher synthesis of IL-6	GG
CRP (rs1205)	T/T - lower production of CRP C/C and C/T - higher production of CRP	CT
SOD2 (rs4880)	C/C and T/C - higher activity of SOD2 enzyme T/T - lower activity of SOD2 enzyme	TT
CAT (rs1001179)	C/C - higher activity of CAT enzyme T/T and C/T - lower activity of CAT enzyme	CT
GPX1 (rs1050450)	C/C - higher activity of GPX1 enzyme T/T and C/T - lower activity of GPX1 enzyme	CT
COL5A1 (rs12722)	C/C - produces COL5A1 with adequate structure C/T and T/T - produces COL5A1 with altered structure	CT

Note: Presentation of all genes, interpretation of each genotype according to literature and athlete’s genotype to Trainability and Adaptation Profiles.

Based on this initial assessment, we have defined the target events for the training period in order to plan the periodization of the strength capacities to be developed, with the main event of the period being the Open Water World Championship Trials in November 2023.

### 2.2 Methodology

**FIGURE 1:**
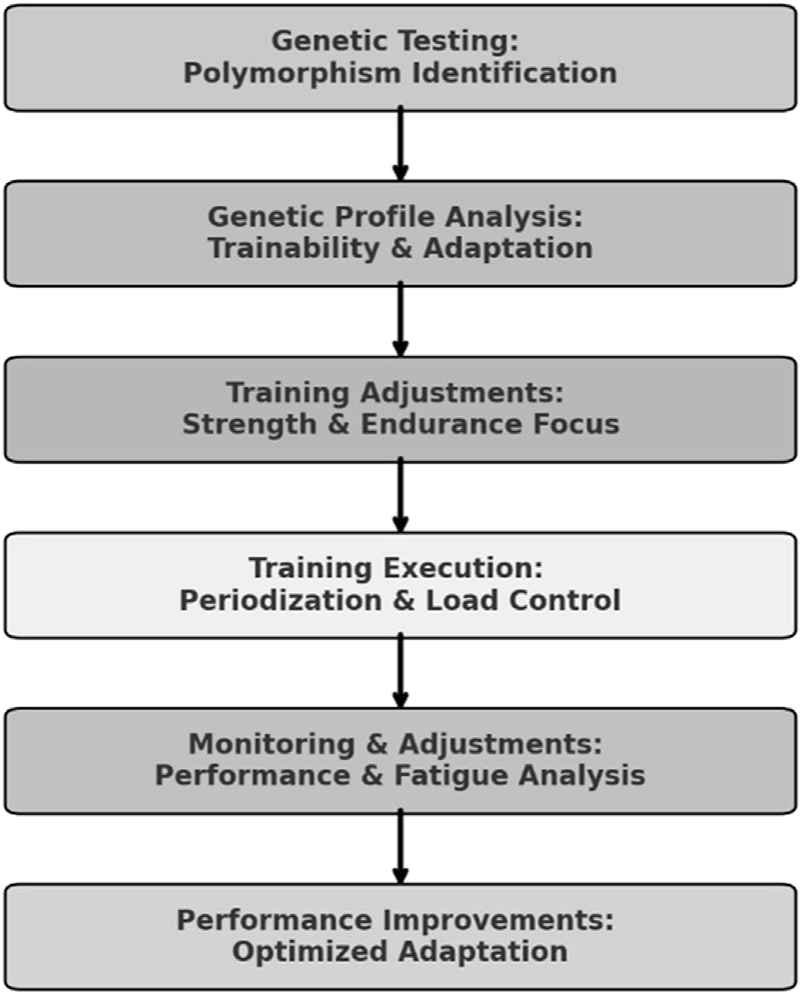
Genomic-Based Training Decision-Making Framework. Note: This flowchart represents the structured decision-making process used to tailor the athlete’s training based on genomic insights. The process begins with genetic testing and profile analysis, which classifies polymorphisms into Trainability & Adaptation categories. Based on this information, training adjustments are made to optimize strength and endurance development. The implementation phase includes periodization and load control, followed by continuous monitoring and adjustments based on performance and fatigue responses. The final outcome is optimized adaptation, contributing to improved athletic performance.

Based on the athlete’s genomic profile, we decided to make two main adaptations: one for specific training in the water and one for physical training. For the aquatic training, we decided to increase the frequency of high-volume and high-intensity stimuli. Although the athlete showed a training profile with a greater potential for short and intense stimuli, his adaptation profile indicated a higher potential for long stimuli. In physical training, we initially focused on developing strength endurance to support this change in water training stimulus. In the second phase, we focused on neuromuscular activation (maximal strength) and muscular power (explosive strength), in alignment with the athlete’s genomic profile, and this was always followed by the development of strength resistance. As this is a case study, we have not presented a statistical analysis, but rather descriptive data. Table 2 presents the anthropometric characteristics of the athlete throughout the year. Table 3 presents the minimum and maximum values for each capacity, as well as the percentage change observed during the training period, calculated with respect to the strength quality being developed in each corresponding phase. The structured decision-making framework used to integrate genetic insights into training is illustrated in Figure 1.

**TABLE 2:** Changes in the athlete’s anthropometric profile over the training period.

Month	Weight (kg)	Height (cm)	IMC (kg/cm^2^)	Fat Mass (%)	Lean Mass (%)	Skinfold (Ʃ)	Body Density (UA)
January	69.5	168.5	24.5	17.4	42.7	107	1,065
June	67.2	168.5	23.7	17.1	42.9	97	1,069
July	66.9	168.5	23.6	16.5	43.1	94	1,069
October	67.3	168.5	23.7	15.2	43.9	82	1,073
December	65.7	168.5	23.1	14.6	44.1	82	1,073

Note: Anthropometric profile based on weight, height, IMC, fat mass, Lean Mass and Body Density from Bioimpedance analysis.

**TABLE 3:** Minimum, maximum, and delta gain in strength capacity over the year.

	Minimum	Maximum	Delta %
Maximum Strength	92.5 ± 38.6	116.25 ± 56.18	25%
Power Strength	56.5 ± 42.49	81.5 ± 61.69	45%
Strength Resistance	31.81 ± 21.56	71.08 ± 49.27	138%

Note: Minimum and Maximum values of all exercises are represented in Mean and Standard Deviation while Delta of the variation between Maximum and Minimum is represented in percentage.

## 3 Results

Table 4 shows when each strength capacity (strength resistance - SR, maximum strength - MS and muscle power - MP) was trained within the proposed periodization. The protocol for each capacity varied during the periodization and followed the basic principles of strength training: SE involved the largest volumes, typically in circuit format; MS involved the smallest volumes and usually the heaviest loads; MP involved moderate loads, but always at the highest speeds. In addition, in all training sessions, the athlete was instructed to perform the exercises at maximum speed, i.e., as fast as possible. The tables with the loads for every exercise during the whole training are on the complementary files.

**TABLE 4:** Strength capacity periodization over the year.

	Strength resistance	Maximum strength	Power strength
April	X		
May	X	X	
June	X	X	
July	X	X	
August	X		
September	X		
October			X
November		X	X
December	X		X
January		X	

Note: Periodization of strength training represented by X on each month the strength capacity was trained.

### 3.1 Training Load Monitoring and strength periodization

To ensure an optimal balance between training adaptations and injury risk, we systematically monitored the athlete’s training load over the course of the intervention. The chronic training load (CTL), which represents long-term training exposure, and the acute-to-chronic workload ratio (ACWR), which provides insight into short-term workload spikes, were key parameters in our assessment. The recommended ACWR range for injury risk mitigation is 0.8–1.3, with values below this threshold potentially leading to detraining and values above increasing the risk of non-functional overreaching and injury.

### 3.2 Training load Trends and periodization strategy

**FIGURE 2:**
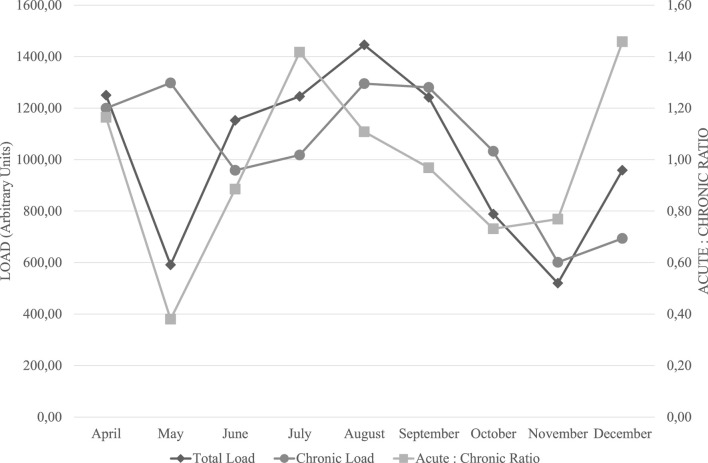
Longitudinal Monitoring of Training Load and Acute-to-Chronic Workload Ratio. Note: This figure illustrates the variations in total training load, chronic training load, and the acute-to-chronic workload ratio (ACWR) over the monitored period. Total load (diamond markers) and chronic load (circular markers) are represented in arbitrary units (AU) on the left y-axis, while ACWR (square markers) is plotted on the right y-axis. The recommended safe range for ACWR (0.8–1.3) is considered to minimize injury risk and optimize performance adaptation (Gabbett, 2016). The fluctuations observed indicate planned training adjustments, including intentional overload periods (July, December) and tapering phases (May, November) aligned with competition goals.

Over the monitored period, CTL remained relatively stable, with fluctuations in acute workload aligning with key phases of the training program. Notably, during May and November, ACWR dipped below 0.8 (0.38 and 0.73, respectively), reflecting intentional reductions in training stress related to tapering strategies. Conversely, in July (1.42) and December (1.46), ACWR exceeded the 1.3 threshold, indicating phases of intensified training stimulus. These peaks aligned with targeted strength training phases aimed at maximal strength (July, November) and power development (October to December), as a deliberate approach to overload prior to key competitions. These variations in total load, chronic load, and acute-to-chronic workload ratio (ACWR) throughout the training period are visually represented in Figure 2. Throughout the periodization plan, strength endurance was prioritized in the early months (April to September), likely to enhance fatigue resistance and facilitate adaptations to the athlete’s genetic predisposition for endurance-related metabolic pathways. Maximal strength training was introduced in May, June, July, and November, serving as a foundation for subsequent power-focused training in October, November, and December. This structured transition aligns with established principles of neuromuscular adaptation, where increased force production capabilities precede power expression.

### 3.3 Implications for performance and fatigue management

The observed fluctuations in training load metrics suggest that the athlete was exposed to varying training intensities while remaining mostly within the safe ACWR range. Notably, the pre-competition taper in November coincided with a lower training load (CTL: 520 AU, ACWR: 1.27), potentially contributing to improved race performance at the Open Water World Championship Trials. Furthermore, the peak in ACWR in July (1.42) reflects a planned overload phase that supported later improvements in power output. The integration of genomic-informed training with systematic load monitoring appears to have facilitated progressive adaptations without excessive risk of overtraining. While ACWR values exceeded 1.3 in certain months, the athlete did not report significant fatigue symptoms or training disruptions, suggesting adequate recovery strategies were in place.

## 4 Discussion

The athlete finished in the top ten at the Open Water World Championship Trials in April 2023, with a total time of 2 h, 2 min, and 37 s, 12 seconds behind the first-placed athlete. At the trials for the second Open Water World Championships in November 2023, our target event, the athlete placed second overall with a time of 1 h, 50 min, and 56 s, just one second behind the first-placed athlete. However, the athlete secured first place among the nationals at the championship event, where the top two swimmers qualified for the World Championships. He finished 12 seconds ahead of the third-place swimmer, who had previously outperformed him by 3 seconds in April.

To achieve such results, we divided the information about the athlete’s genotype into two different groups: Trainability and Adaptation. The genes assigned to the “Trainability” group provided information on how the athlete’s body might react acutely to various physical capacities during training sessions, while the genes assigned to the “Adaptation” group provided information on how the athlete’s body might adapt longitudinally to these stimuli.

We opted for an integrated analysis of the function of the proteins expressed by these genes rather than a score based on the sum of the potentials of the different genotypes, as is usually done (Yvert et al., 2016). That’s because we were aware that genetic information follows the principle of biological individuality, and this would not be achieved if we categorized the athlete into a qualitative profile (Ruiz et al., 2009). Instead, analyzing each genotype based on the physio-metabolic function of the gene allowed for a more nuanced understanding of the athlete’s unique genetic makeup.

### 4.1 Trainability profile

Of all the genes analyzed, we believe that ACTN3 is the most important. It is the gene that provides the most conclusive results and helps us to prescribe the specific training variables for strength development (El Ouali et al., 2024; Ferreira et al., 2024). This is because among all twenty polymorphisms analyzed, ACTN3 is the only gene that codes to produce a structural protein (alpha-actinin 3) found only in type II muscle fibers (MacArthur and North, 2004). The R allele refers to the production of the α-actinin-3 protein, while the X allele does not produce it. Phenotypically, the ACTN3 gene is directly related to muscle fiber type distribution (Vincent et al., 2007), and individuals expressing the R allele tend to experience less muscle damage and reduced strength loss after intense exercise (Del Coso et al., 2017). In contrast, individuals with the XX genotype have been shown to have twice the risk of muscle injury compared to those with RX or RR genotypes (Moreno et al., 2020).

Knowing that our athlete had the RR genotype, we started to investigate the other polymorphisms in the trainability profile. Basically, there are two types of training to analyze (continuous training and interval training), and performance in each of these domains depends on metabolic adaptations mediated by these polymorphisms. We found that the athlete has a greater potential for short bursts of high intensity due to possible phenotypic characteristics associated with the polymorphisms of the MCT1, AGT, BDKRB2, NOS3 and ADRB2 genes, despite the opposite characteristic represented by the polymorphism of the ACE gene.

Our approach relied on various studies that revealed the phenotypic potential of each genotype in relation to the athlete’s internal load control throughout the training period. The analysis of interval training began with the potential acute response to training with lactic anaerobic characteristics, considering first the MCT1 genotype. Heterozygosity would potentially result in the athlete taking up a lower amount of lactate by the muscle itself (Fedotovskaya et al., 2014), but with training we could increase the amount of this transporter (Dubouchaud et al., 2000) and thus improve lactic-anaerobic endurance. Reduced uptake of lactate and H+ ions by muscle may increase heart and respiratory rates during the buffering process. At this stage, we started to investigate the phenotypic response that the other genes (AGT, BDKRB2, NOS3 and ADRB2) might show during the recovery phases after a lactic-anaerobic stimulus or during a continuous aerobic stimulus.

The polymorphisms of the AGT, BDKRB2, NOS3 and ADRB2 genes, taken together would suggest something that we had already observed in practice: The athlete tends to need more time to recover from intense stimuli (heart rate remains high) or to reach a steady-state during continuous stimuli, even during the race. According to our analysis, the ADRB2 gene polymorphism could influence a lower initial adrenergic response (Sarpeshkar and Bentley, 2010), thereby hindering oxygen delivery to active muscles and maintaining a greater reliance on anaerobic metabolism. The longer recovery time between stimuli or to compensate for energy demands could be explained by higher angiotensinogen production (AGT gene) (Zmijewski et al., 2016), which is associated with potentially lower bradykinin receptor production (BDKRB2 gene) and lower nitric oxide synthase enzyme activity (NOS3 gene) (Saunders et al., 2006). The only positive aspect of this axis is the ACE gene polymorphism, which may have lower angiotensin-converting enzyme activity, favoring an aerobic response (Voroshin and Astratenkova, 2008) and reducing bradykinin degradation (Wong et al., 2016).

Thus, we hypothesize that lactic anaerobic stimuli would be easier for the athlete to perform than continuous aerobic stimuli. To examine the best distribution of these training sessions and the associated physical capacities, we created a second profile, which we call adaptation.

### 4.2 Adaptation profile

Within this profile, we have analyzed some genes that are important from the perspective of training control, but not necessarily for monitoring and controlling training load. The first genes analyzed belong to the peroxisome proliferator-activated receptor (PPAR) family, as they are related to lipid metabolism in response to exercise (Thomas et al., 2012; Nakamura et al., 2014; Grygiel-Górniak, 2014; Chen et al., 2022). Since we had favorable genotypes for all genes of the PPAR family, we hypothesized that adaptations to aerobic training could be facilitated even if the training sessions were more demanding.

A second point we analyzed was the propensity for inflammation in response to training, mainly due to the athlete’s recent injury history. To this end, we examined the polymorphisms of the TNF-α, IL6, CRP, SOD2, CAT and GPX1 genes, with only the TNF-α gene showing a favorable genotype (potentially lower expression of the protein). This profile, combined with the need to increase the athlete’s performance for a long race such as the Open Water Marathon (10 km), led us to choose a higher load of physical training for the development of strength resistance. In this way, we limited the muscle stress and micro-injuries that could lead to an increase in CRP (Isaacs et al., 2019), reducing the acute phase of tissue repair, reducing the possibility of TNF-α mediated inflammatory response and reducing the induction of oxidative stress (Baumert et al., 2016). On the other hand, this decision could enhance the antioxidant response mediated by SOD2, CAT and GPX1 (Ogino et al., 2021; Powers et al., 2022).

A final point analyzed was the genotype of COL5A1 and its influence on joint flexibility and mobility (Ogino et al., 2021). Additionally, we considered evidence suggesting a genetic predisposition to rotator cuff injury, particularly the presence of variants such as rs71404070, which has been associated with a 29% increased risk of rotator cuff tears (Roos et al., 2017). This information helped guide our preventive and compensatory strategies during physical preparation. Again, due to the pre-existing injury, we took precautions to use fewer complex exercises with a wide range of motion. In this way, we were able to understand the actual needs of the athlete based on their genomic profile and ensure the integrity of the principle of biological individuality, and as concluded by Keegan et al. (Keegan et al., 2017), we believe that this case report can help improve the connection between research and practice in the introduction of genetic information into sports training planning.

## 5 Practical applications

The practical application of genetic information in sports science has been widely discussed in the literature, with several studies examining the influence of genetic variants on athletic performance and injury susceptibility. Previous research efforts have successfully identified genetic markers associated with muscle fibre composition, VO2 max, anaerobic threshold, injury risk, and recovery efficiency. However, the direct integration of this genetic data into real-time training modifications has remained largely unexplored. Unlike previous studies that primarily identify genetic predispositions, our approach actively applies this information to guide real-world training decisions. By adjusting the training protocol based on the athlete’s genomic profile, we enable more precise load management, targeted injury prevention strategies, and optimized recovery periods—tailored to the athlete’s unique physiological needs. This distinction is crucial, as our study demonstrates the feasibility of using genetic insights to directly shape an athlete’s training program, rather than solely identifying predispositions. While the generalization of this approach to larger cohorts remains a challenge, this study provides a strong proof of concept for the future integration of genomics into elite sports training methodologies.

## 6 Ethical considerations

The integration of genetic information into sports performance and training programs remains a topic of ongoing debate. While the potential of genomic data to personalize training and injury prevention strategies is increasingly recognized, current expert guidelines emphasize the need for ethical considerations and scientific validation before widespread adoption. The FIMS 2019 consensus statement (Tanisawa et al., 2020) highlights the importance of ethical data use, privacy protection, and the necessity of large-scale, reproducible studies to support the clinical utility of genetic testing in sports​. Furthermore, McAuley et al. (2023) stress that genetic testing in sports should prioritize talent inclusion and individualized athlete development rather than exclusionary selection processes​. In alignment with these recommendations, our study aims to demonstrate a responsible and evidence-based approach to incorporating genetic information into training protocols, ensuring that genetic insights are used to optimize, rather than limit, an athlete’s development.

## 7 Limitations of the study

While this study provides an innovative application of genomic data in personalized training interventions, it is important to acknowledge its inherent limitations as an n = 1 case study. The findings presented here may not be directly generalizable to other sports or athlete populations, as genetic influences on performance are context-dependent and influenced by sport-specific demands, training background, and environmental factors. Additionally, while our approach demonstrates a practical framework for integrating genetics into elite training, the individual response to genomic-based training adjustments may vary among athletes. Future research should aim to replicate these findings in multi-case studies and controlled cohort designs to further evaluate the effectiveness and reproducibility of genomics-based training personalization across different sports and athlete populations.

## 8 Conclusion

We concluded that the study of the characteristics of genomic information in high performance sports can help to understand the strengths and weaknesses of athletes and thus design better training programs that truly respect the principle of biological individuality. Furthermore, we can add that strategies such as TGS may not be suitable for application in training because they cannot assess the interaction between genes to the extent of extracting information that is applicable in the daily practice of the coach, as shown in this work.

## Data Availability

The datasets presented in this study can be found in online repositories. The names of the repository/repositories and accession number(s) can be found in the article/Supplementary Material.
