# Supplementary material for: Genomic information in the decision-making process for the training of a high-performance brazilian swimmer: a case report
Source: Front Genet. 2025 Apr 15;16:1544178. doi: 10.3389/fgene.2025.1544178 (PMC12037596; doi:10.3389/fgene.2025.1544178)
Supplement: Supplementary file 1 [file DataSheet1.pdf]

CARE Checklist for Case Report Submission

1. **Title**
   - The manuscript's title is "Genetic information in the decision-making process for the training of a high-performance Brazilian swimmer: A Case Report."
2. **Key Words**
   - Genetics, swimming, periodization, high-performance, case report.
   - *Line*: 11
3. **Abstract**
   - **Introduction**: Although numerous genetic variations have been associated with athletic profiles and performance, limited literature exists on the practical integration of this information into daily training, particularly for elite athletes.
     - *Line*: 13-15
   - **Patient's Main Concerns and Clinical Findings**: This case study involves a 23-year-old elite open water marathon swimmer whose primary goal was to qualify for the Absolute World Championships in 2024. The athlete presented a consistent competitive history but sought optimized training strategies to enhance performance and secure a top position in national and international competitions.
     - *Line*: 16-20
   - **Primary Diagnoses, Interventions, and Outcomes**: Twenty genetic polymorphisms were analyzed to inform and structure the athlete's training plan over one year. The interventions included tailored training regimens that aligned with the athlete's genetic predispositions, aiming to maximize physiological responses and recovery. The outcome was an improvement in athletic performance, highlighted by a top finish among compatriots and qualification for the Absolute World Championships.
     - *Line*: 20-24
   - **Conclusion**: With this case report we demonstrated that genetic-based training may be an effective strategy to assist sports professionals in making training planning decisions for high performance athletes. This approach may provide valuable support for sports professionals in decision-making related to training strategies for elite competitors.
     - *Line*: 24-27
4. **Introduction**

- This case stands out by applying genetic analysis of twenty polymorphisms to long-term training plans for an elite swimmer, a novel approach supported by limited literature.
- *Line*: 36-42

5. **Patient Information**
    - The athlete was a 23-year-old male, a competitive open water swimmer for 10 years. Relevant medical history included tendinopathy in the left shoulder with partial tendon tears in late 2022.
    - *Line*: 29-35
6. **Clinical Findings**
    - Genetic-based training increase athletes' strength (maximal, power and resistance) and reduces the impact of a pre-existent shoulder injury.
    - *Line*: 31-35; 81-87
7. **Timeline**
    - initial injury (late 2022), genetic testing (early 2023), training modifications, and competition results (April and November 2023).
    - *Line*: 31-37; 64-66; 89-96
8. **Diagnostic Assessment**
    - **Methods**: Genetic testing via real-time PCR, analysis of 20 polymorphisms.
        - *Line*: 55-61
    - **Challenges**: Ensuring personalized training plans aligned with genetic data.
        - *Line*: Discussed in "Trainability Profile," lines 109-148
    - **Diagnosis**: Predisposition for high-intensity performance, with potential for aerobic challenges.
        - *Line*: "Trainability Profile" section, lines 109-148
    - **Prognostic Characteristics**: Favorable outcomes for endurance with structured training.
        - *Line*: Lines 149-171, continued into "Adaptation Profile" section
9. **Therapeutic Intervention**
    - **Types**: Adjusted strength training to align with genetic predispositions, focusing on endurance, power, and maximum strength.

        - *Line*: 68-79
    - **Details**: Phased training from strength endurance to maximal power.
        - *Line*: 89-107
    - **Modifications**: Adapted based on genetic results to optimize performance.
        - *Line*: 109-171

10. **Follow-up and Outcomes**
    - **Outcomes**: Improved strength metrics
        - *Line*: 80-87
    - **Follow-up Tests**: Regular assessments of anthropometric and performance data.
        - *Line*: Mentioned in "Results" section, lines 80-87
    - **Adverse Events**: None reported.
        - *Line*: No explicit mention; inferred from overall results.
11. **Discussion**
    - Strengths include personalized training, and limitations involve generalizability. The report contributes to applying genetic data to sports science.
        - *Line*: Lines 88-179
    - **Conclusion**: Integrating genetic data into training can support individualized approaches for elite athletes.
        - *Line*: 180-186
12. **Patient Perspective**
    - "The results of the genetic test not only clarified genetic aspects but also impacted my training routine. After two months, I began to notice improvements in my training and, consequently, in competitions. This contributed to my qualification for the world championship."
        - *Line*: 203-205 (Acknowledgments)
13. **Informed Consent**
    - Confirm that informed consent was obtained from the athlete to report this case.
        - *Line*: 54
